# A Cost Evaluation of the Georgia Stroke and Heart Attack Prevention Program

**Published:** 2005-12-15

**Authors:** David B Rein, Diane Orenstein, Roberta T Constantine, Hong Chen, Patricia Jones, J. Nell Brownstein, Rosanne Farris

**Affiliations:** Research Triangle Institute International, Waltham, Mass; Division for Heart Disease and Stroke Prevention, National Center for Chronic Disease Prevention and Health Promotion, Centers for Disease Control and Prevention; Research Triangle Institute International, Waltham, Mass; Research Triangle Institute International, Waltham, Mass; Georgia Division of Public Health, Chronic Disease Prevention and Health Promotion Branch, Atlanta, Ga; Division for Heart Disease and Stroke Prevention, National Center for Chronic Disease Prevention and Health Promotion, Centers for Disease Control and Prevention, Atlanta, Ga; Division for Heart Disease and Stroke Prevention, National Center for Chronic Disease Prevention and Health Promotion, Centers for Disease Control and Prevention, Atlanta, Ga

## Abstract

**Introduction:**

Hypertension is a leading cause of stroke, coronary artery disease, heart attack, and heart and kidney failure in the United States, all of which contribute to the rising costs of health care. The Georgia Stroke and Heart Attack Prevention Program is an education and direct service program for low-income patients with hypertension. This project evaluated the cost-effectiveness of the program compared with the following two alternative scenarios: no treatment for high blood pressure and the typical hypertension treatment received in the private sector nationwide (usual care).

**Methods:**

We estimated the preventive treatment costs and number of adverse health events averted (hemorrhagic and ischemic stroke, heart disease, and kidney failure) associated with the Georgia Stroke and Heart Attack Prevention Program in two Georgia health districts. We used program cost and service usage data obtained from the Georgia Department of Human Resources and probabilities and costs of expected adverse events published in peer-reviewed sources. We compared program costs and number of expected adverse health events averted with those expected from 1) no preventive care and 2) usual care for high blood pressure.

**Results:**

The Georgia Stroke and Heart Attack Prevention Program was less costly and resulted in better health outcomes than either no preventive care or usual care. Compared with no preventive care in the two districts, the program was estimated to result in 54% fewer expected adverse events; compared with usual care, the program was estimated to result in 46% fewer expected adverse events. Combining the costs of preventive treatment with the costs of expected adverse events, the Georgia Stroke and Heart Attack Prevention Program cost an average of $486 per patient annually, compared with average annual costs of $534 for no care and $624 for usual care.

**Conclusion:**

Maintaining a publicly financed stroke and heart attack prevention program is more cost-effective and results in greater health benefits than other plausible scenarios. Because the benefits of this program accrue to both the state and federal governments through reduced Medicaid and indigent care expenditures, both the state and federal governments have a financial incentive to support the program.

## Introduction

Hypertension is a leading cause of stroke, coronary artery disease, heart attack, and heart and kidney failure in the United States. Currently, 50 million Americans have hypertension and another 45 million have prehypertension (blood pressure of 120–139 mm Hg [systolic] or 80–89 mm Hg [diastolic]) (1). More than 70% of U.S. adults with hypertension do not have it under control (2,3). Hypertension is particularly common among African Americans, who have a 30% higher prevalence of hypertension than whites (1). As might be expected, African Americans experience hypertension-related deaths at younger ages than whites and have higher rates of stroke, left ventricular hypertrophy, and heart attack (3). Some but not all of these differences are explained by the lower socioeconomic status (SES) of African Americans, because lower SES is also strongly related to uncontrolled blood pressure (4). As many as 30% of all deaths among African American men and 20% of all deaths among African American women can be attributed to high blood pressure (5).

Aggressive treatment of hypertension, which usually involves medication, significantly decreases the risk of coronary artery disease, congestive heart failure, stroke, and resulting disability. For example, a 12-point to 13-point reduction in blood pressure can lower the risk of heart attack by 21%, stroke by 37%, and total cardiovascular deaths by 25% (6). Results of recent large hypertension trials demonstrated that inexpensive thiazide-type diuretics are superior in preventing one or more major forms of cardiovascular disease (7). Unfortunately, low-income individuals without prescription drug coverage are significantly more likely to skip doses to save money or make their hypertension medication prescriptions last longer. In one recently observed population, systemic hypertension was adequately controlled among only 38% of those who paid for their medication themselves (8).

The Georgia Stroke and Heart Attack Prevention Program (SHAPP) is an education and direct service program for low-income patients with hypertension. The program is based on the Chronic Care Model, a framework for identifying the essential elements of a health care system and involving patients in their own care (9). SHAPP patient services are provided through the county health departments and include screening, referral to physicians, diagnosis, and treatment. Treatment protocols are based on Joint National Committee on Prevention, Detection, Evaluation, and Treatment of High Blood Pressure recommendations (10). Individual patients are assigned to nurses who act as case managers. SHAPP nurses then coordinate the wide array of treatment services including physical and family history assessments, diagnostic testing, lifestyle counseling and education, medication, and patient monitoring (follow-up visits for medication, blood pressure assessment, and any needed testing) as stipulated by the SHAPP protocol. Information on patient medical and family history, physical characteristics, and risk factors is collected. Diagnostic testing is done to determine baseline and follow-up blood pressure levels. Lifestyle counseling includes advice on diet, weight, smoking cessation, alcohol consumption, and physical activity and information about signs and symptoms of stroke and heart attack. Nurses track medication side effects and monitor patients to ensure they keep clinic appointments and adhere to medication schedules. SHAPP also supplies prescription drugs at low or no cost according to a treatment protocol.

According to the Centers for Disease Control and Prevention's (CDC's) Behavioral Risk Factor Surveillance System (BRFSS), 1.7 million Georgians had hypertension in 2004. Of those, 469,800 were low income, uninsured, or underinsured and were potentially eligible for the SHAPP program. (Eligibility is based on both income and hypertension severity.) However, given the small enrollment in SHAPP (15,819 clients in fiscal year 2003), there is likely a much a greater need in Georgia for SHAPP services than the program is currently able to meet.

Reducing hypertension can lead to marked reductions (10) in the risk of several high-consequence adverse events such as hemorrhagic stroke (11), ischemic stroke, heart disease (12), and kidney failure (13). However, one recent study suggests that less intensive interventions that rely only on patients to manage their own hypertension care are relatively ineffective (14). Comparatively, SHAPP is a higher-intensity intervention guided by the premise that providing low-cost preventive services to medically indigent patients provides benefits to patients and savings to the state. SHAPP patients who control their blood pressure could reduce their risks for adverse events, thus prolonging and improving the quality of their lives and lowering the annual medical costs borne by the state for high-cost hospital care and procedures. Although advocates of SHAPP have long suspected that the program results in cost savings, the association among SHAPP services, patient outcomes, and medical costs has never been formally evaluated. In this article, we discuss a limited, first-time evaluation of the costs and benefits of SHAPP to determine whether this promising practice results in enhanced patient health and reduced medical costs for the state.

## Methods

### Selection of districts

We collected administrative data on the costs and hypertension control outcomes of SHAPP in two Georgia health districts with high rates of blood pressure control for fiscal year 2003 (July 1, 2002–June 30, 2003). Blood pressure *control* is defined by the Georgia Department of Human Resources (DHR) as a reading of less than 140/90 mm Hg, based on the average of at least two blood pressure readings taken on the most recent visit.

The selection and number of the districts studied was guided by several factors. The primary goal of the study was to examine the critical components of SHAPP in sites with high success rates so that lessons could be shared with other hypertension control programs. In addition to analyzing costs, the full study included focus groups with patients, interviews with clinic and administrative staff, and an examination of the medical records. Time and funding limitations allowed for only two districts to be studied. The two districts were selected based on their success in controlling hypertension, use of different computer systems, geographic diversity between districts, demographic diversity among counties within the districts, and the mix of patients managed solely by the health department with patients managed jointly by the health department and private physicians. Although the characteristics of SHAPP districts and clinics vary widely, the intention is for SHAPP to perform at the same level in all districts. This analysis represents the upper boundary of the potential effectiveness of the SHAPP program.

### Impact of SHAPP compared with plausible alternatives

After examining administrative data for each district, we extrapolated the number of adverse health events — hemorrhagic stroke (11), ischemic stroke, heart disease (12), and kidney failure (13) — that would be expected given the level of blood pressure control within each district, and we assigned costs to these events. We then compared cost and health outcomes of SHAPP with two simulated plausible alternatives: 1) no care and 2) the typical treatment received in the private sector nationally (referred to as *usual care*). We chose no care to represent the lower boundary that Georgia's SHAPP patients would receive in the absence of the program, and we chose usual care to represent the upper boundary. If SHAPP were eliminated, its patients could be expected to receive no care (the worst-case scenario) or usual care (the best-case scenario).

Patients who received no care would be expected to have no costs related to blood pressure control, but they would also be expected to have a higher number of adverse events. Our analysis evaluated whether the number of expected adverse events prevented by SHAPP was sufficient to justify the additional cost of SHAPP preventive treatment.

Next, we compared SHAPP patients with a scenario in which patients who had characteristics identical to SHAPP patients received usual care. SHAPP patients and patients who received usual care would be expected to differ in the following ways: 1) cost of treatment, 2) level of hypertension control outcomes, and 3) the probability of receiving treatment. Nationally, only 58% of people with hypertension receive any regular preventive care, compared with 100% of SHAPP patients, assuming SHAPP patients seek care and are eligible for the program (1). This analysis evaluated whether SHAPP resulted in less costly treatment as well as fewer expected adverse health events than usual care. Advocates have suggested that SHAPP is both less expensive for and more effective at controlling blood pressure than care provided in private settings because SHAPP uses evidence-based protocols whereas private providers may substitute alternative protocols; SHAPP uses fewer new, more costly (and not necessarily more effective) prescription drugs; and SHAPP substitutes nurse practitioners for physicians to manage patient care. SHAPP could be more beneficial than usual care by offering full coverage to all patients who are eligible and seek treatment, by providing services at lower costs, or by achieving better outcomes.

### SHAPP data and costs

We examined typical direct and indirect SHAPP program costs using methodology recommended by the National Panel on Cost-Effectiveness Analysis in Health and Medicine convened by the U.S. Public Health Service (15). Total program costs include the cost of services (e.g., diagnostic testing, patient visits), medications, and overhead costs. Overhead costs include personnel costs (e.g., salary, benefits) and operating costs (e.g., pharmacy, clinical). Total annual SHAPP clinical costs were supplied for two selected counties in District 1 and District 2. For each district, data were obtained from one county in which the study occurred and in another county selected randomly.

The Georgia DHR supplied the following data for the selected counties: 1) the number of patients treated and the percentage of patients who achieved controlled blood pressure; 2) the costs of prescription drugs, postage, and overhead; and 3) the cost of clinical services. To estimate clinical costs per patient, we divided the annual total costs per county by the total number of SHAPP patients treated in each county. We assumed that the costs identified in the selected counties were representative of the overall per-patient costs in each health district.

The percentage of SHAPP patients treated with prescription drugs was calculated by dividing the number of patients who received any prescription drug in each district by the number of patients in that district. The cost per patient for prescribed drugs was calculated by dividing the total annual prescription drug cost in each district by the number of patients who received any drugs. Postage costs per patient were calculated by dividing aggregate postage costs (to mail prescriptions to SHAPP clinics) in each district by the number of patients in each district. Government overhead costs for SHAPP statewide were provided by Georgia DHR for all SHAPP patients statewide. These costs included personnel costs (salary and benefits) and operating costs (pharmacy and clinical). This total cost was converted to a per-person cost by dividing it by the number of statewide SHAPP participants, and this cost was then applied to patients in the respective districts. SHAPP's annual treatment cost per patient was calculated as the sum of per-patient clinical, prescription drug, postage, and state overhead costs.

### Comparison treatment costs

For comparison purposes, estimates of the number of patients receiving treatment and the expected level of hypertension control for patients who receive treatment were obtained from reported national hypertension trends derived from the third National Health and Nutrition Examination Survey (NHANES III) (1). Because no exact definition exists of what treatment in the private sector entails, we assumed that such treatment was based on the Seventh Report of the Joint National Committee on Prevention, Detection, Evaluation, and Treatment of High Blood Pressure (JNC 7) guidelines, which would be comparable to SHAPP treatment (3). Therefore, we defined usual care (for people who received treatment) as the average number of annual hypertension visits for SHAPP participants (3.71 visits), combined with the same drug treatment used by SHAPP patients in District 1.

The cost of usual care was estimated by multiplying the average number of annual hypertension visits for SHAPP participants (3.71 visits) by the Centers for Medicaid and Medicare Services (CMS) Medicare reimbursement rate for office visits ($80). To this we added the average per-person drug costs observed in the SHAPP program. We assumed no postage or state overhead costs for usual care. Because not all patients with hypertension receive care, we then multiplied this total per-patient cost for those who receive care by the estimated proportion of patients who receive any treatment (1). Table 1 presents a comparison of costs and outcomes for patients in District 1 and District 2 and for patients nationally.

We noted that prescription costs per patient were higher in District 1. On further examination of the data, we found that even though drug costs per type of prescription were consistent statewide, District 1 used a greater quantity of some drugs such as hydrocortothiazide, hydralazine, and fosinopril. In contrast, per-patient clinical services were more costly in District 2. Although most reported procedure codes are the same in both districts, the data show that some differences in service use exist. Overall, patients in District 2 used more clinical services such as phone consultation, preventive counseling, and laboratory test reviews than patients in District 1. Also, the cost per procedure was routinely more expensive per unit of service in District 2 than District 1. Focus groups with patients and key informant interviews with administrators and staff in both districts indicated that each SHAPP district offers the same basic set of services. The observed differences in costs between the two districts studied, however, indicate that SHAPP implementation varies among districts; the technology used to capture and report cost and usage data may vary as well.

### Outcomes

We defined SHAPP effectiveness as the proportion of patients with controlled blood pressure in each health district, based on statistics from Georgia DHR annual reports. Using these reported levels of blood pressure control, we then estimated the number of adverse events expected in each health district based on the results of a published statistical model by Flack and colleagues (12). The model was designed to estimate the annual probability of hemorrhagic and ischemic stroke and heart disease for individuals in three categories of blood pressure treatment and control: 1) treated and controlled, 2) treated but uncontrolled, and 3) untreated and uncontrolled (12). We used this model to make estimates because time and funding limitations prevented us from observing adverse outcomes or measuring associated costs directly. The Flack study was selected for modeling purposes because it provided the most recent and comprehensive information related to the SHAPP study. It examined the effect of inadequate blood pressure control on selected cardiovascular disease outcomes and analyzed related costs for the U.S. population with hypertension. In addition, the study developed a sophisticated model, provided incidence rates for cardiovascular disease morbidity and mortality, and integrated hypertension statistics from NHANES III and cost estimates for stroke, congestive heart failure, and myocardial infarction (12). NHANES III was conducted in 1999–2000; the published results were the most recent available at the time of the Flack study.

We calculated the cost-effectiveness of SHAPP by comparing two other treatment possibilities — no treatment and usual care — based on expected adverse outcomes observed in the absence of a public program. The proportions of the U.S. population with treated and controlled, treated but uncontrolled, and untreated and uncontrolled blood pressure were taken from a published analysis of NHANES III surveillance data for 1999–2000 (1) (Table 2). The probability of hemorrhagic stroke based on treatment and control of blood pressure was taken from the published results of a randomized controlled study of more than 45,000 participants in the Netherlands that provided population-based estimates; the goal of this study was to examine the outcomes (i.e., number of strokes) associated with insufficient treatment of hypertension (11). The probability of ischemic stroke was derived by applying the ratio of ischemic strokes to hemorrhagic strokes identified in the 2001 Medical Expenditure Panel Study (MEPS) (16) to the probabilities of hemorrhagic stoke identified in the Netherlands study. The probability of kidney failure was derived from two separate studies of hypertension-related adverse events, the second of which studied hypertension-related renal failure (13,17). Rates of heart disease and the costs of each expected adverse event were obtained from the Flack simulation model of cardiovascular disease associated with uncontrolled blood pressure (12). Costs for stroke and heart attack reported in the Flack study represent estimates of inpatient and outpatient costs during 1 year after the adverse event. Inpatient costs represent the majority of costs and were obtained from the National Inpatient Profile (a database derived from the National Hospital Discharge Survey); outpatient costs included typical follow-up care, medications, laboratory tests, and office visits (12). The cost of treating congestive heart failure was obtained from the economic burden-of-illness estimates from the American Heart Association (12).

## Results

The SHAPP program achieved blood pressure control rates of 68.1% in District 1 and 59.7% in District 2 (Table 1). The average control rate for all SHAPP districts is 54%, with a range of 41% to 68% (data not shown). The comparative national control rate was 53% for patients in treatment, translating to a 31% control rate for all patients nationally when accounting for individuals who do not seek treatment (Table 2) (1). Annual preventive treatment costs per patient were $132.36 in District 1 and $260.39 in District 2. The average number of clinical services between the two districts and the same pharmaceutical care that was used in District 1 would cost $322 per patient in the private sector. However, because only 58% of patients with hypertension nationally receive preventive treatment, the estimated national annual per-patient cost for treated patients with hypertension ($187.04) was between the annual costs per patient in the two districts.

Because SHAPP achieved higher blood pressure control rates and offered care to all patients who were eligible and sought treatment, SHAPP patients in both districts were expected to experience lower rates of hemorrhagic stroke, ischemic stroke, heart disease, and kidney failure compared with both other treatment scenarios (no care and usual care) (Table 3). Patients in District 1 were expected to experience roughly 10 fewer expected adverse events than if they had received no treatment and seven fewer than if they had received usual care. Patients in District 2 were expected to experience roughly 30 fewer expected adverse events than if they had received no treatment and 21 fewer than if they had received usual care.

The differences in the number of expected adverse outcomes translated into substantial differences in costs among the three scenarios. Total expected annual costs for SHAPP patients, including both preventive treatment and care related to expected adverse outcomes in District 1, were estimated at $289,617 for no treatment, $323,095 for usual care, and $209,800 for SHAPP treatment. In District 2, costs were estimated at $870,451 for no treatment, $971,070 for usual care, and $848,254 for SHAPP treatment (Table 4). In each county, SHAPP was the least expensive of the three treatment scenarios. Total expected costs for SHAPP patients in District 1 were 27.5% below the costs of no treatment, and 35.1% below the costs of treatment offered only through the private sector. In District 2, where treatment costs were higher, total costs of SHAPP were 2.6% below the costs of no treatment and 12.7% below the costs of treatment offered only through the private sector. When examining both districts to provide a more global picture of SHAPP, we found that implementation of the SHAPP program resulted in both lower costs and greater potential health benefits than either of the alternative treatment scenarios. SHAPP saved costs and provided greater health benefits when compared with both no treatment for hypertension and usual care (Table 5).

SHAPP costs differed between the two health districts. Table 1 shows that although District 1 reported lower costs per patient overall than District 2, District 1 prescribed medications to a greater proportion (94%) of clients than District 2 (63%). District 1 also paid more for medications ($49.56 per patient) than District 2 ($15.19 per patient). In contrast, District 1 used fewer clinical services per patient (8.0) than District 2 (12.6) and paid less for the services.

## Discussion

SHAPP is an education and direct service program that appears to save costs for the state of Georgia. SHAPP resulted in lower costs and better health outcomes than either no treatment or treatment offered at the average level expected nationally. For the two districts examined, SHAPP was found to be preferable to the other two options because it resulted in both better blood pressure control levels (which are expected to translate into fewer adverse health events), lower treatment costs for those who receive treatment, and lower overall costs per eligible patient. These results were supported by blood pressure control rates of 68.1% in District 1 and 59.7% in District 2. Both these rates exceed the national average control rate for patients in treatment (53%) and for all patients with hypertension, including the untreated (31%). The average control rate for SHAPP during this study period was 54%. Although the SHAPP districts that were evaluated had higher control rates, we believe that these rates can be reached by other districts.

SHAPP still saved costs when expected adverse outcomes were considered, although the cost savings covered a narrow range of direct medical costs. Had we included lost productivity and deaths associated with the expected adverse events that SHAPP prevented, the benefits of SHAPP compared with the benefits of other treatment options would likely appear even more substantial. This cost analysis was one element of an initial program evaluation that also included both medical record review and focus groups with patients as well as key informant interviews with administrative and clinical staff. This evaluation also identified important components that contribute to high blood pressure control rates: intense patient monitoring, follow-up, access to medication, and counseling.  

This study is limited by several factors. First, because of funding and time constraints, only two districts were examined. Also, districts with high blood pressure control rates were analyzed because the primary objectives of the study were to examine program components that contributed to successful blood pressure outcomes and to communicate results to other hypertension programs. Limited information on adverse events among SHAPP patients was available, and no actual outcomes in the population were observed. All expected adverse events were inferred from the medical literature using the probability of adverse events based on different levels of blood pressure control. The conclusions are accurate to the extent that populations observed in other studies reflect the characteristics of the SHAPP population. Populations in other studies used for this analysis vary in their similarity to the SHAPP population. When selecting parameter values for the model, we balanced demographic similarity with data completeness, fit of the parameter, and size of the study. For example, Klungel et al used data from a study of more than 45,000 individuals and thus had the statistical power to observe differences that would be missed by other smaller studies. In addition, Klungel et al examined precisely the measure we sought: the probability of stroke given uncontrolled and controlled hypertension.

Second, medical costs for private-sector care were estimated based on service usage observed in SHAPP. Thus, the results may overestimate the effectiveness of the SHAPP statewide. A better approach for estimation would include direct observation of the costs of preventive services in the private sector and comparison of private-sector costs with SHAPP costs. In addition, a more comprehensive approach would be to examine SHAPP on a statewide basis, including districts with varying rates of success. Future models should use more complicated simulations that vary the number of assumptions.

On the other hand, the costs assigned in this study were conservative, particularly prescription costs. A more precise estimate of private-sector costs would likely make SHAPP appear more cost-effective because SHAPP provides protocol-driven, evidence-based treatment, and most treatment is provided by nurses instead of physicians. We have no way of knowing how SHAPP patients would use services in the absence of the program, so we must rely on hypothetical scenarios for comparison. Finally, because we used data from two higher-performing SHAPP districts, this study identified the upper range of program effectiveness. However, hypertension control rates representing all SHAPP districts (ranging from 41% to 68%) exceed the rates of hypertension control observed nationally in NHANES (31%).

This study evaluated the costs and benefits of SHAPP based on observed data on program costs and outcomes and on similar data published in the medical literature translated into adverse events in other settings. These findings show that SHAPP treatment is more cost-effective than no treatment or treatment offered only through the private sector. Compared with the two other plausible scenarios tested, SHAPP resulted in the lowest medical costs and the best patient health outcomes. Given these conclusions, we hypothesize that SHAPP's full coverage of patients is preferable to both no patient care and the average amount of care expected nationally both in terms of costs and health outcomes. It is important to view these results in the context of the growing expense of health care and the importance of implementing prevention programs that are successful and reduce costs.

However, it is also important to keep the limitations of the study in mind. Promising practices at the state level too often are left unexplored because of a lack of funding or an inability to acquire data. This evaluation provides preliminary evidence of the effectiveness of an intervention based on the Chronic Care Model to control hypertension among disadvantaged individuals, but limited time and resources led to a simple simulation that used data from a limited number of locations and relied on several assumptions. We recommend more extensive and more formal evaluations of SHAPP and other hypertension interventions based on the Chronic Care Model to better understand how these interventions work and to more precisely measure program success.

Because SHAPP costs are borne by the taxpayer and at least some portion of private care is paid for by consumers, it is important to consider who would bear the costs of caring for adverse outcomes if SHAPP were eliminated. First, because patients in SHAPP are indigent, they would be far more likely to receive no care than the average amount of care received nationally if the program were eliminated. This analysis suggests that such a change would result in a substantial increase of expected adverse events and deaths. Because most SHAPP patients do not carry private insurance, the higher cost of caring for these adverse events would likely fall on already overextended public hospitals, the state Medicaid program, and federally funded indigent care programs. Thus, the elimination of SHAPP would likely result in higher costs for both the state and federal governments, making it financially prudent for Georgia to maintain the program. The higher costs of not providing care due to the occurrence of adverse events far exceed the costs of treating hypertension and preventing those events. More importantly, SHAPP provides access to vital services for indigent Georgians to address this critical, life-threatening health issue.

## Figures and Tables

**Table 1:** Costs and Outcomes for Patients in Districts 1 and 2 of the Georgia Stroke and Heart Attack Prevention Program Compared With National Values

**Cost or Outcome**	**District 1 (N = 543)**	**District 2 (N = 1632)**	**National (Usual Care)**
Percentage of patients with controlled blood pressure	68.1	59.7	31^a^
No. of clinical services per patient per year	8.0	12.6	Not estimated
Clinical cost per patient treated, $ per year	79.58	244.59	296.80
Percentage of treated patients using prescription drugs	94	63	78^b^
Prescription costs per patient prescribed, $ per year	49.56	15.19	32.73^b^
Postage cost per patient, $ per year	0.44	0.10	0
State overhead costs per patient, $ per year	5.61	5.61	0
Percentage of patients receiving preventive treatment	100	100	58
Annual preventive treatment cost per patient, $ per year	132.36	260.39	187.04^a^

a We assumed that only 58% of patients with hypertension receive preventive treatment in the absence of a public program, as suggested by the literature (1), and estimated that the hypertension control rate for patients receiving treatment was 53% (1).

b We assumed that these values were equal to the average of the SHAPP districts.

**Table 2:** Differences in Annual Expected Rates of Adverse Health Events Among Americans With Treated and Controlled, Treated but Uncontrolled, and Untreated Hypertension

	**Category of Hypertension Care**			**Cost Per Event (in 2001 Dollars)**

	**Treated and Controlled, %**	**Treated but** ** Uncontrolled, %**	**Untreated, %**	
Rates among Americans with hypertension^a^	31.0	27.4	41.6	Not applicable

**Adverse event**						

Hemorrhagic stroke^b^	0.13	0.16	0.28	26,000
Ischemic stroke^c^	0.54	0.66	1.12	26,000
Heart disease^d^	0.70	0.90	1.20	12,300
Kidney failure^e^	0.22	0.27	0.77	2,822

a Source: Hajjar and Kotchen (1)

b Sources: Klungel et al (11) and Flack et al (12).

c We assumed that the ratio of hemorrhagic stroke to ischemic stroke is 1:4 (16).

d Source: Flack et al (12).

e Sources: Tierney et al (13), Perneger et al (17), Smith et al (18).

**Table 3:** Expected Number of Annual Adverse Health Events Associated With District 1 and District 2 Patients in the Georgia Stroke and Heart Attack Prevention Program (SHAPP) Under Three Treatment Scenarios

**Adverse Event**	**District 1 (N = 543) Treatment Scenario**			**District 2 (N = 1632) Treatment Scenario**		

	**None**	**National (Usual Care)**	**SHAPP**	**None**	**National (Usual Care)**	**SHAPP**
Hemorrhagic stroke	1.52	1.09	0.69	4.57	3.28	2.12
Ischemic stroke	6.08	4.43	2.76	18.28	13.32	8.46
Heart disease	6.52	5.23	3.66	19.58	15.73	11.26
Kidney failure	4.19	4.80	1.12	12.59	14.44	3.42
**Total**	**18.31**	**15.56**	**8.23**	**55.02**	**46.77**	**25.27**

**Table 4:** Annual Estimated Costs of Expected Adverse Health Events Associated With District 1 and District 2 Patients in the Georgia Stroke and Heart Attack Prevention Program (SHAPP) Under Three Treatment Scenarios

**Adverse Events and Total Costs**	**Estimated Costs, $**					

	**District 1 (N = 543) Treatment Scenario**			**District 2 (N = 1632) Treatment Scenario**		

	**None**	**National (Usual Care)**	**SHAPP**	**None**	**National (Usual Care)**	**SHAPP**

**Adverse event**						

Hemorrhagic stroke^a^	39,530	28,391	17,953	118,810	85,330	55,009
Ischemic stroke^a^	158,122	115,203	71,815	475,238	346,245	220,037
Heart disease^a^	80,146	64,385	45,002	240,883	193,510	138,577
Kidney failure^b^	11,818	13,554	3,157	35,520	40,736	9,675

**Total costs, $**						

Total cost of adverse events	289,617	221,532	137,929	870,451	665,821	423,297
Total cost of preventive treatment	0	101,563	71,871	0	305,249	424,957
Total combined costs (event plus preventive treatment)	289,617	323,095	209,800	870,451	971,070	848,254

a Data from Flack et al (12).

b Data from Smith et al (18).

**Table 5:** Annual Expected Adverse Health Events and Estimated Costs Associated With Patients in the Georgia Stroke and Heart Attack Prevention Program (SHAPP) Under Three Treatment Scenarios

**Event or Cost**	**District 1 and District 2 Combined (N = 2175) Treatment Scenario**		

	**None**	**National (Usual Care)**	**SHAPP**
Total no. of adverse events	73.32	62.33	33.50
Incremental benefit compared with no preventive treatment	Not applicable	11.00	39.82
Total cost of adverse events, $	1,160,068	887,353	561,227
Total cost of preventive treatment, $	0	406,812	496,828
Combined costs (event plus preventive treatment), $	1,160,068	1,294,165	1,058,055
Total combined costs per patient, $	534	595	486
